# Prevalence and Distribution of *Salmonella* in Water Bodies in South America: A Systematic Review

**DOI:** 10.3390/microorganisms13030489

**Published:** 2025-02-22

**Authors:** Makarena Sofia Gonzalez Reyes, Rayana Santos Araujo Palharini, Felipe Ferreira Monteiro, Salvador Ayala, Eduardo A. Undurraga

**Affiliations:** 1 Center for Bioinformatics and Integrative Biology, Facultad de Ciencias Biológicas, Universidad Andrés Bello, Santiago 8370146, RM, Chile; makarenagonzalezreyes@gmail.com; 2 Departamento de Prevención de Riesgos y Medio Ambiente, Universidad Tecnológica Metropolitana, Santiago 8330383, RM, Chile; 3 Departamento de Ciências Atmosféricas e Climáticas, Universidade Federal do Rio Grande do Norte, Natal 59078-970, RN, Brazil; felipefmonteiro@gmail.com; 4 Centro de Epidemiología y Políticas de Salud (CEPS), Universidad Del Desarrollo, Las Condes, Santiago 7610658, RM, Chile; salvadorayala@udd.cl; 5 Escuela de Gobierno, Pontificia Universidad Católica de Chile, Macul, Santiago 7820436, RM, Chile; eundurra@uc.cl; 6 Research Center for Integrated Disaster Risk Management (CIGIDEN), Macul, Santiago 7820436, RM, Chile

**Keywords:** Chile, climate change, waterborne diseases, *Salmonella*, remote sensing

## Abstract

The presence of *Salmonella* in rivers, lakes, or beaches in South America represents a challenge to public health and aquatic ecosystems. This review explores the distribution, prevalence, and the main factors contributing to the survival and spread of Salmonella, including wastewater discharge, agricultural runoff, and climatic variables such as high temperatures and precipitation. These factors also facilitate the distribution of multidrug-resistant strains in water. The review is based on bibliographic searches in various databases, focusing on *Salmonella* species, South American countries, and types of water bodies. Predominant serovars include *S.* Enteritidis and *S.* Typhimurium, with *S.* Typhi and *S.* Panama frequently detected in Chile, *S.* Enteritidis in Argentina, and *S.* Typhimurium in Brazil. Less common serovars, including *S.* Dublin and *S.* Paratyphi B, were identified, along with subspecies such as diarizonae and houtenae. These findings highlight the role of environmental, physicochemical, and anthropogenic factors influencing *Salmonella* dynamics. The review identifies research gaps, advocating for further studies to better understand the interactions between Salmonella, climate change, and human activity. Strengthening surveillance and mitigation strategies is crucial to protect water resources and public health in South America.

## 1. Introduction

Water bodies are essential resources, playing a critical role in sustaining human communities and natural ecosystems [1,2]. These water bodies support essential activities such as drinking water supply, agriculture, hydropower, recreation, and aquatic biodiversity [3,4,5,6]. However, rapid urbanization, intensive agriculture, and the lack of efficient waste management systems have placed growing pressure on water quality in the region [7,8]. Pathogen contamination of water bodies poses a major threat, affecting both human health and ecosystems [9,10].

To address these challenges, South American countries have implemented various monitoring and mitigation strategies. These include microbiological and physicochemical monitoring, remote sensing, and hydrological modeling to predict pathogen dispersion [11]. Mitigation efforts focus on improving wastewater treatment, promoting sustainable agriculture, and restoring aquatic ecosystems. However, the lack of standardized methods, real-time data, and effective enforcement limits their impact [11]. Strengthening monitoring frameworks and targeted strategies is essential to controlling waterborne pathogens [11].

Among microbiological contaminants, *Salmonella* stands out as one of the most significant pathogens due to its public health and environmental impact [12,13]. This bacterial genus, widespread in terrestrial and aquatic environments, causes salmonellosis and typhoid fever, affecting millions worldwide each year [14]. *Salmonella* enters water bodies through multiple pathways, including untreated wastewater, agricultural runoff carrying fertilizers and manure, and improper disposal of industrial waste [15], with risks exacerbated in regions with limited sanitation infrastructure and poor waste management [16,17].

Beyond its direct impact on public health, *Salmonella* can disrupt aquatic ecosystems [18] by altering local microbial communities and reducing biodiversity [19,20]. In aquatic environments, *Salmonella* can persist and proliferate under various environmental conditions, including elevated temperatures and high levels of organic matter [12,21]. Additionally, antibiotic use in agriculture and aquaculture promotes the growth of *Salmonella* and the emergence of multidrug-resistant strains, compounding the issue [10].

Climate factors, such as precipitation and elevated temperatures, are key variables in the distribution and incidence of *Salmonella* in water bodies [12,22]. Precipitation can transport contaminants from terrestrial areas into water bodies, increasing bacterial loads, while higher temperatures prolong *Salmonella* survival in the environment, enhancing its capacity for dissemination [23,24,25]. These dynamics are particularly relevant in the context of climate change, which is altering precipitation and temperature patterns across many regions of South America, further complicating control efforts [26,27].

Understanding how climatic factors and local contamination dynamics influence the incidence and distribution of *Salmonella* in water bodies is essential for developing more effective monitoring and mitigation strategies. Assessing the prevalence of *Salmonella* in different water bodies across South America will help identify incidence patterns and areas of higher vulnerability and inform the design of policies aimed at reducing health risks and protecting aquatic ecosystems.

## 2. Materials and Methods

This bibliographic review was conducted using a combination of search terms applied across PubMed, Web of Science, ScienceDirect, and Google Scholar to identify studies on *Salmonella* in water bodies across South America. The search strategy was informed by previous reviews to ensure comprehensive literature coverage.

The search strategy was divided into three search components (SC): SC1 *Salmonella* species, including search terms specifically targeting the genus and species level to ensure the review captured the largest number of studies. SC2 country of origin, including search terms for South American countries (Argentina, Bolivia, Brasil, Chile, Colombia, Ecuador, Guyana, Paraguay, Perú, Suriname, Uruguay, and Venezuela), aiming to refine the search to studies conducted in these regions to gather specific data. SC3 water body types, including an extensive list of water-related terms in both English and Spanish, such as Bay (Bahía), Beach (Playa), Brook (Riachuelo), Drainage basin (Cuenca hidrográfica), Estuary (Estuario), Glacier (Glaciar), Gulf (Golfo), Lagoon (Laguna), Lake (Lago), Ocean (Océano), Port (Puerto), Puddle (Charco), Reservoir (Embalse), Ria (Ría), River (Río), River delta (Delta fluvial), River mouth (Desembocadura), Sea (Mar), Ship canal (Canal de navegación), Strait (Estrecho), Stream (Arroyo), Swamp (Pantano), Tidal marsh (Marisma), Well (Pozo), and Wetland (Humedal), to capture studies across diverse aquatic environments.

**Figure 1:**
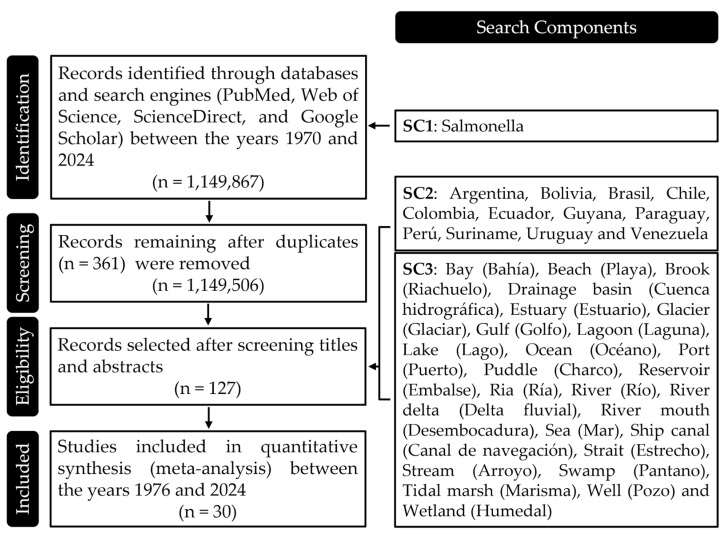
Search Components Used in the Review Process. This figure provides a detailed breakdown of the search components employed during the literature review. Each component was carefully crafted to capture a comprehensive range of studies relevant to the presence of *Salmonella* in water bodies across South America. The components are categorized by species identification, geographical focus, and types of water bodies, ensuring a thorough and targeted search strategy that covers the essential aspects of the research topic.

Given the large number of articles identified under SC1, SC2 and SC3 were applied to article titles and abstracts to refine results. The specific search terms and criteria for each component are detailed in Figure 1. A manual review of the selected articles was conducted to ensure relevance and a manageable dataset. The selection covered publications from 1970 to 2024. Additionally, manual searches were performed by reviewing the reference lists of the initially selected articles. This step helped to identify any further studies that met the inclusion criteria but may not have been captured in the database search.

A total of 30 studies were included: 1 study from Venezuela, 2 from Colombia and Perú, 3 from Bolivia, 6 from Chile, 7 from Brazil, and 10 from Argentina (Brazil and Chile shared one study).

The variability in the methodologies used to detect the presence of *Salmonella* prevented the application of comparative statistical analyses. For this reason, the review focused solely on the presence or absence of *Salmonella* in water bodies, without assessing relative abundance or other quantitative factors. This limitation arose because the selected studies employed different detection approaches, making their results not directly comparable. Additionally, many articles analyzed various types of water bodies within the same country, further complicating the identification of general patterns.

To ensure transparency and rigor in the review process, the Preferred Reporting Items for Systematic Reviews and Meta-Analyses (PRISMA) guidelines were followed [28]. This framework helped standardize the selection and reporting of studies, ensuring a structured and comprehensive bibliographic review.

## 3. Results

### 3.1. Subsection: Diversity and Clinical Relevance of Salmonella Serovars in Aquatic Environments Across South America

*Salmonella* is a genus of gram-negative bacteria belonging to the family Enterobacteriaceae, widely distributed in terrestrial and aquatic environments [12]. This genus includes two main species: *Salmonella enterica* and *Salmonella bongori*, with the former being responsible for most clinically significant infections [29]. Within *S. enterica*, six subspecies have been identified: enterica (I), salamae (II), arizonae (IIIa), diarizonae (IIIb), houtenae (IV), and indica (VI), which together comprise more than 2600 serovars [30]. These serovars differ in their specific combinations of somatic (O) and flagellar (H) antigens [31]. The subsp. enterica includes the majority of clinically and environmentally relevant serovars, being the most frequently isolated in human and animal infections [32]. Among the serovars, *S.* Typhi and *S.* Paratyphi B are associated with systemic diseases such as typhoid and paratyphoid fever, whose transmission is often linked to the consumption of contaminated water [33]. In contrast, serovars like *S.* Enteritidis and *S.* Typhimurium have been detected in water but are also widely associated with zoonotic and foodborne infections, affecting both humans and animals [33]. Additionally, these studies have reported less common serovars, such as Dublin, which primarily affect cattle [34]. Moreover, within the subsp. diarizonae, the serovar 16:z10:e,n,x,z15 has been identified, primarily infecting frogs and snakes [35]. These findings highlight the diversity of *Salmonella* in aquatic environments and its potential role in bacterial transmission.

Table 1 provides a summary of the serovars of *Salmonella enterica* subsp. enterica identified in various water bodies across South America, along with their respective hosts and associated diseases. These serovars have been isolated from a variety of aquatic environments, such as streams, lakes, and rivers. The primarily affected hosts include humans, swine, cattle, and poultry, causing diseases such as gastroenteritis, one of the most common clinical manifestations. Among the most relevant serovars of *S. enterica* subsp. enterica identified are:

Enteritidis: One of the most frequent serovars, associated with infections in both humans and poultry [36]. In humans, it primarily causes gastroenteritis accompanied by fever, and in severe cases, it can progress to septicemia [13]. In poultry, it generally presents asymptomatically, although it can affect egg quality, making poultry an important reservoir and a source of transmission to humans through contaminated food [37].

Dublin: This serovar primarily affects swine and cattle. It is known to cause gastroenteritis, fever, and septicemia, as well as abortions in infected cows [34]. In humans, although infections are less common, they tend to be severe, particularly in immunocompromised individuals, highlighting their importance as a zoonotic pathogen [34].

Typhimurium: This serovar infects a wide range of hosts, including humans, pigs, cattle, and horses [38]. It is one of the leading causes of salmonellosis in humans, manifesting symptoms such as gastroenteritis, fever, and, in severe cases, septicemia [39]. In animals, it can cause intestinal and systemic infections that compromise their health and productivity, particularly in intensive agricultural systems [40,41].

Paratyphi B: This serovar has been isolated from humans and is associated with gastritis and paratyphoid fever [42]. Although this systemic disease is less severe than typhoid fever, it represents a significant risk, particularly in areas with poor water quality, where transmission through contaminated water is more likely [43].

In addition to the serovars of the subsp. enterica identified in surface waters (rivers, canals, streams, ponds, and reservoirs, among others), several serovars belonging to other subspecies were also found. Within the subsp. diarizonae, the serovars 35:r:z, 38:(k):z35, 50:r:z, 61:l,v:z, P:k:z35, 18:i:z, 18:k:z, 18:z10:e,n,x,z15, 48:i:z, 58:k:z, 61:i:z, 65:(k):z, and 16:z10:e,n,x,z15 were identified; the latter primarily infects frogs and snakes [35]. Meanwhile, in the subsp. houtenae, the serovars 40:z4,z24:-, 43:z4,z23:-, R:z4,z24:-, and 16:z4,z32:- were found, with the latter associated with humans as its main host [44]. Additionally, a representative of the subsp. salamae was identified, corresponding to the serovar 42:r:-.

**Table 1:** Serovars of *Salmonella enterica* subsp. *enterica* isolated from water bodies in South America, their main hosts, and associated diseases.

	Host *					Diseases		
Serovar	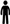	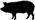	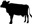	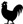	Other	GE	Other	Ref.
Agona								[45]
Anatum					Birds			[46]
Bareilly								[47]
Braenderup								[47]
Corvallis								[48]
Derby					Birds		Septicemia	[33]
Dublin					Sheep		Abortion, Fever, Septicemia	[33]
Enteritidis					Wild rodents		Fever, Septicemia	[33]
Give					Ruminants		Splenic abscess	[49]
Heidelberg								[50]
Infantis								[51]
Javiana					Various animals			[52]
Kentucky								[53]
London								[53]
Manhattan								[54]
Mbandaka								[55]
Meleagridis								[56]
Montevideo								[57]
Newport								[56]
Ohio							Bone abscess	[58]
Oranienburg					Various animals			[59]
Paratyphi B							Fever, Septicemia	[33]
Rissen								[60]
Typhi							Fever, Septicemia	[33]
Typhimurium					Horses		Fever, Septicemia	[33]

* The silhouettes shown in the table represent the categories Humans (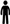), Swine (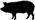), Poultry (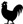), and Cattle (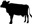), while the abbreviation GE stands for gastroenteritis, a disease affecting the gastrointestinal tract.

### 3.2. Distribution of Salmonella in South American Bodies of Water

**Figure 2:**
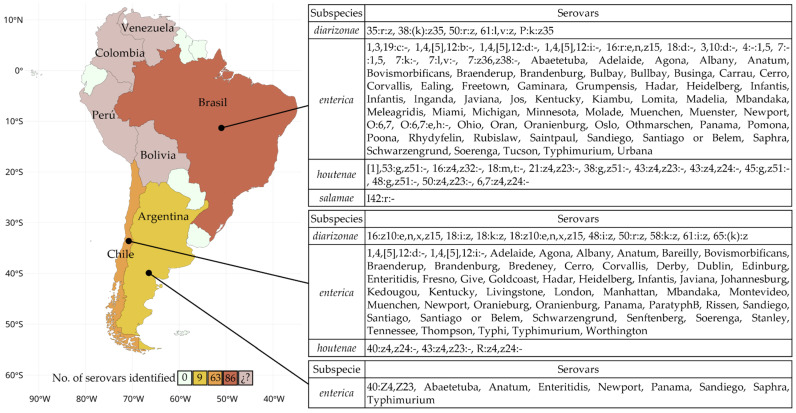
Map of South America representing the number of *Salmonella* serovars identified in water bodies by country. The colors indicate the number of reported serovars. In the case of Bolivia, Colombia, Perú, and Venezuela, (¿?) *Salmonella* was detected, but only at the genus level.

The contamination of water bodies by *Salmonella* represents a critical issue in South America. This pathogen is widely detected in rivers, lakes, and estuaries, which are essential for human activities such as drinking water, recreation, and agriculture. Its presence degrades water quality, threatens food security, and disrupts aquatic ecosystems. The diversity of *Salmonella* serovars recorded in different countries (Figure 2, Table S1), reflects the variety of contamination factors and the need for continuous monitoring.

In Argentina, *Salmonella* has been detected in major hydrological systems, including the Luján, San Luis, Negro, and de la Plata rivers. The latter, used for consumption, recreation, and fishing [61], is contaminated by direct wastewater discharge and the transport of pollutants during precipitation events [6,62,63]. It presents high levels of bacteria resistant to multiple drugs, posing a significant risk to public health and local ecosystems [6]. In the Luján River, *S.* Enteritidis [64] was predominant, commonly associated with poultry farming and transmission through contaminated food [65]. Multiple serovars were detected in the San Luis River, including Enteritidis, Newport, Panama, Sandiego, and Typhimurium [66]. Their presence could be linked to the discharge of domestic wastewater and agro-industrial effluents, which contribute organic matter and nutrients, favoring their survival and dispersion [18]. In addition to these large river systems, the presence of *Salmonella* has also been identified in other water bodies across the country, such as the Maldonado Canal [67], Lake Argüello [68], Napostá Stream [67], and La Choza [63] (Table 2).

In Bolivia, the La Paz River exhibits heavy pollution from untreated urban wastewater discharges, particularly from densely populated areas and hospitals [69,70]. *Salmonella* has been identified in 83–92% of water samples collected from impacted sites, highlighting its high prevalence in specific areas of the basin [69]. The Holguín site, located 17.4 km downstream from Incachaca, is situated in a densely populated urban area, right next to a hospital compound, making it a critical contamination point. Mecapaca, located 40 km from Incachaca in the agricultural lowlands, is another area impacted by wastewater discharges, as is the Jillusaya River, an urban lateral tributary of the La Paz River [69]. In these areas, *Salmonella* is among the most prevalent enteric pathogens in surface waters. Isolated strains exhibit resistance to multiple antibiotics, including ampicillin, trimethoprim-sulfamethoxazole, nalidixic acid, cefoxitin, and tetracycline [69,71], suggesting the possible dissemination of resistance genes from urban and hospital sources [69]. Moreover, the presence of genes associated with extended-spectrum beta-lactamases (*blaCTX-M*) could indicate the potential horizontal transfer of resistance genes among different bacterial species in the aquatic environment [71] (Table 2 and Table S1).

In Brazil, studies have primarily focused on rivers, where *S.* Typhimurium has been primarily identified in the Arrudas and Onça rivers [72], along with the serovars Madelia, Panama, and Saintpaul in the Jaguaribe River [16]. These serovars are associated with aquaculture environments, such as shrimp farms, and the discharge of untreated wastewater. In particular, *S.* Typhimurium can tolerate a wide range of physicochemical conditions in effluent water [73], contributing to its persistence in these environments. Additionally, along with the serovar Panama, it is one of the most prevalent serotypes isolated from swine in Brazil [74,75,76,77] (Table 2).

In Rio de Janeiro and Paraíba, various subspecies of *Salmonella* have been identified, each with distinct characteristics and specific serovars. The subsp. diarizonae, naturally found in reptiles, includes serovars such as 35:r:z, 38:(k):z35, 50:r:z, 61:l,v:z, and P:k:z35 [78]. Meanwhile, the subsp. houtenae, which inhabits the intestinal tract of reptiles and establishes an endophytic relationship with the natural environment [44,79], is represented by serovars such as [1], 53:g,z51:-, 16:z4,z32:-, 18:m,t:-, 21:z4,z23:-, 38:g,z51:-, 43:z4,z23:-, 43:z4,z24:-, 45:g,z51:-, 48:g,z51:-, 50:z4,z23:-, and 6,7:z4,z24:- [78]. Finally, the subsp. salamae, with a more restricted distribution and less frequently associated with virulence in humans or animals [80], includes the serovar 42:r:- [78].

In Chile, contamination by *Salmonella* is widespread across various regions and areas with different land uses, including rural, peri-urban, and urban zones. In the Metropolitan Region, the Zanjón de la Aguada and the Mapocho and Maipo rivers have been identified as significant sources of *Salmonella* contamination, including serovars Panama [14], Typhi [81], and Typhimurium [81]. In the Zanjón de la Aguada, which is highly polluted with industrial waste and untreated wastewater [82], concerning levels of *S.* Typhi were detected in water used for irrigation in nearby agricultural areas [81,83]. This pattern is also observed in the Maipo River, which supplies approximately 90% of the irrigation needs of the Metropolitan Region, and in the Mapocho River [84], where agricultural activities and septic systems significantly contribute to microbiological contamination [83,85]. Studies have identified the prevalence of *Salmonella* spp. in 50% of irrigation water samples collected from rivers and canals in these areas [81,83] (Table 2). In rural areas such as Calera de Tango and Isla de Maipo, serovars such as Mbandaka, Montevideo, Heidelberg, Infantis, and Panama were detected [85], along with specific groups like C2 and C3 [85]. In peri-urban areas, such as Melipilla and Talagante, serovars like Agona, Corvallis, Newport, Enteritidis, and Livingstone were identified, highlighting the diversity of serotypes in these locations [83,85] (Table 2). In rural and peri-urban zones such as Paine, Peñaflor, and María Pinto, serovars Brandenburg, Santiago, and Anatum were identified [85], along with others like Infantis and Senftenberg, showing a high prevalence in agricultural and livestock systems. In the Maule and Metropolitan regions, subspecies such as diarizonae and houtenae have been documented [78,85], as well as an extensive variety of serovars from subsp. enterica, including Adelaide, Agona, Albany, Anatum, Bovismorbificans, Braenderup, Brandenburg, Derby, Dublin, Enteritidis, Infantis, Kentucky, Montevideo, Newport, Panama, Paratyphi B, Schwarzengrund, Senftenberg, and Typhimurium [83,85]. These findings reflect a relationship between human activities, such as waste management and the use of wastewater in agriculture, and the spread of *Salmonella*, posing a significant risk to public health and food safety [81,83] (Table 2).

In Colombia, *Salmonella* has been documented in both freshwater and coastal environments. In the Bogotá River, which receives untreated wastewater from Bogotá and other localities [86], high concentrations of *Salmonella* were found, ranging between 10^5.3^ and 10^8.4^ CFU/L by direct isolation [86]. On the Atlantic coast beaches, such as Palmarito, Puerto Colombia, Salgar, and Santa Verónica, *Salmonella* spp. was detected in 14% of water samples [87], mainly in areas near channels and streams discharging untreated wastewater. Urban beaches like Salgar and Puerto Colombia showed higher prevalence, attributed to poor waste management and intense human activity in these areas [87] (Table 2).

In Perú, studies in the Huatanay River revealed the presence of *Salmonella* associated with high levels of antibiotic residues, such as amoxicillin and ceftriaxone [17]. These substances, originating from livestock practices and domestic and industrial discharges, not only favor the survival of *Salmonella* in the environment but also increase antimicrobial resistance in the detected strains [17] (Table 2 and Table S1).

**Table 2:** Summary of *Salmonella* presence in South American water bodies: this table highlights the diversity of species and serovars, as well as their geographical distribution.

Country	Body of Water	Geographical Location	Species	Subspecies	Serovar	Ref.
Argentina	Canal	Maldonado	*Salmonella* spp.			[67]
	Downstream	Merlo	*Salmonella* spp.			[63]
	Lake	Argüello	*Salmonella* spp.			[68]
	River	Club de Regatas Beach	*Salmonella* spp.			[68]
	River	Río de la Plata	*Salmonella* spp.			[6]
	River	Luján	*Salmonella enterica*	enterica	Enteritidis	[64]
	River	Luján	*Salmonella* spp.			[88]
	River	Negro	*Salmonella* spp.			[68]
	River	San Luis	*Salmonella enterica*	enterica	Enteritidis, Newport, Panama, Sandiego, Typhimurium	[66,89]
	Stream	La choza	*Salmonella* spp.			[63]
	Stream	Napostá	*Salmonella* spp.			[67]
	Stream	Zaimán	*Salmonella enterica*	enterica	Abaetetuba, Saphra, Anatum, Newport, Saphra	[90]
Bolivia	River	Choqueyapu	*Salmonella enterica*	enterica		[71]
	River	Jillusaya	*Salmonella* spp.			[69]
	River	La Paz	*Salmonella enterica*	enterica	O:4	[91]
	River	La Paz (Holguín)	*Salmonella* spp.			[69]
	River	La Paz (Mecapaca)	*Salmonella* spp.			[69]
Brazil	Estuary	Ubicado en São Francisco do Conde	*Salmonella* spp.			[10]
	Estuary	Ubicado en Valença	*Salmonella* spp.			[10]
	Lake		*Salmonella enterica*	enterica	Ealing, Infantis, O:6,7, O:6,7:e,h:-	[92]
	River	Arrudas	*Salmonella enterica*	enterica	Typhimurium	[72]
	River	Camboa Grande	*Salmonella* spp.			[93]
	River	Jaguaribe	*Salmonella enterica*	enterica	Madelia, Panama, Saintpaul	[16]
	River	Jaguaribe	*Salmonella enterica*	houtenae		[16]
	River	Onça	*Salmonella enterica*	enterica	Typhimurium	[72]
	River	São João	*Salmonella* spp.			[23]
	Surface water *	Rio de Janeiro and Paraíba	*Salmonella enterica*	diarizonae	35:r:z, 38:(k):z35, 50:r:z, 61:l,v:z, P:k:z35	[78]
	Surface water *	Rio de Janeiro and Paraíba	*Salmonella enterica*	enterica	Abaetetuba, Adelaide, Agona, Albany, Anatum, Bovismorbificans, Braenderup, Brandenburg, Bulbay, Bullbay, Businga, Carrau, Cerro, Corvallis, Freetown, Gaminara, Grumpensis, Hadar, Heidelberg, Infantis, Inganda, Javiana, Jos, Kentucky, Kiambu, Lomita, Madelia, Mbandaka, Meleagridis, Miami, Michigan, Minnesota, Molade, Muenchen, Muenster, Newport, Ohio, Oran, Oranienburg, Oslo, Othmarschen, Panama, Pomona, Poona, Rhydyfelin, Rubislaw, Saintpaul, Sandiego, Santiago or Belem, Saphra, Schwarzengrund, Soerenga, Tucson, Typhimurium, Urbana	[78]
	Surface water *	Rio de Janeiro and Paraíba	*Salmonella enterica*	houtenae	[1],53:g,z51:-, 16:z4,z32:-, 18:m,t:-, 21:z4,z23:-, 38:g,z51:-, 43:z4,z23:-, 43:z4,z24:-, 45:g,z51:-, 48:g,z51:-, 50:z4,z23:-, 6,7:z4,z24:-	[78]
	Surface water *	Rio de Janeiro and Paraíba	*Salmonella enterica*	salamae	42:r:-	[78]
Chile	Channel	Zanjon de la Aguada	*Salmonella enterica*	enterica	Typhi	[81]
	Municipality	Calera de Tango (rural)	*Salmonella enterica*	enterica	Mbandaka	[85]
	Municipality	Colina (urban)	*Salmonella enterica*	enterica	Montevideo	[85]
	Municipality	Isla Maipo (rural)	*Salmonella enterica*	enterica	Heidelberg, Infantis, Panama	[85]
	Municipality	Isla Maipo (rural)	*Salmonella* sp. Group C2			[85]
	Municipality	Isla Maipo (rural)	*Salmonella* sp. Group C3			[85]
	Municipality	La Florida (urban)	*Salmonella enterica*	enterica	Enteritidis	[85]
	Municipality	La Pintana (urban)	*Salmonella enterica*	enterica	Typhimurium	[85]
	Municipality	Maria Pinto (rural)	*Salmonella enterica*	enterica	Anatum, Mbandaka, Typhimurium	[85]
	Municipality	Melipilla (peri-urban)	*Salmonella enterica*	diarizonae		[85]
	Municipality	Melipilla (peri-urban)	*Salmonella enterica*	enterica	Agona, Corvallis, Newport	[85]
	Municipality	Melipilla (rural)	*Salmonella enterica*	enterica	Corvallis, Enteritidis, Typhimurium	[85]
	Municipality	Paine (rural)	*Salmonella enterica*	enterica	Brandenburg, Santiago, Typhimurium	[85]
	Municipality	Paine (rural)	*Salmonella* sp. Group C4			[85]
	Municipality	Peñaflor (peri-urban)	*Salmonella enterica*	enterica	Infantis, Panama	[85]
	Municipality	Talagante (rural)	*Salmonella enterica*	enterica	Brandenburg, Senftenberg	[85]
	Municipality	Talagante (peri-urban)	*Salmonella enterica*	enterica	Enteritidis, Give, Livingstone, Typhimurium	[85]
	Municipality	Talagante (peri-urban)	*Salmonella* sp. Group C1			[85]
	River	Claro	*Salmonella* spp.			[83]
	River	Lontue	*Salmonella* spp.			[83]
	River	Maipo	*Salmonella* spp.			[83]
	River	Mapocho	*Salmonella enterica*	enterica	Panama, Typhi	[14,81]
	River	Mapocho	*Salmonella* spp.			[83]
	River	Mataquito	*Salmonella* spp.			[83]
	River	Santiago	*Salmonella enterica*	enterica	Agona, Anatum, Bareilly, Bredeney, Derby, Enteritidis, Infantis, Livingstone, London, Oranieburg, Paratyphi B, Senftenberg, Thompson, Typhimurium	[9]
	River	Santiago	*Salmonella* sp. Group K			[9]
	Surface water *	Maule and MR *	*Salmonella enterica*	diarizonae	16:z10:e,n,x,z15, 18:i:z, 18:k:z, 18:z10:e,n,x,z15, 48:i:z, 50:r:z, 58:k:z, 61:i:z, 65:(k):z	[78]
	Surface water *	Maule and MR *	*Salmonella enterica*	enterica	Adelaide, Agona, Albany, Anatum, Bovismorbificans, Braenderup, Brandenburg, Cerro, Corvallis, Derby, Dublin, Edinburg, Enteritidis, Fresno, Give, Goldcoast, Hadar, Infantis, Javiana, Johannesburg, Kedougou, Kentucky, Livingstone, Manhattan, Mbandaka, Montevideo, Muenchen, Newport, Oranienburg, Panama, Paratyphi B, Rissen, Sandiego, Santiago or Belem, Schwarzengrund, Senftenberg, Soerenga, Stanley, Tennessee, Thompson, Typhimurium, Worthington	[78]
	Surface water *	Maule and MR *	*Salmonella enterica*	houtenae	40:z4,z24:-, 43:z4,z23:-, R:z4,z24:-	[78]
Colombia	Beach	Palmarito	*Salmonella* spp.			[87]
	Beach	Puerto Colombia	*Salmonella* spp.			[87]
	Beach	Salgar	*Salmonella* spp.			[87]
	Beach	Santa Veronica	*Salmonella* spp.			[87]
	River	Bogota	*Salmonella* spp.			[86]
Peru	Beach	La Chira	*Salmonella* spp.			[94]
	River	Huatanay	*Salmonella* spp.			[17]
	River	Surco	*Salmonella* spp.			[94]
Venezuela	Lagoon	Grande del Obispo	*Salmonella* spp.			[95]

* Surface water refers to rivers, canals, streams, ponds, dams, and other similar bodies of water, as the specific type of water body was not precisely defined in the study. The abbreviation MR stands for Metropolitan Region.

### 3.3. Factors Influencing the Development of Salmonella in Water Bodies

*Salmonella* is a widely distributed pathogen in the environment, capable of proliferating in rivers, lakes, beaches, and other water bodies due to multiple factors. Key factors include climatic and environmental conditions, such as temperature and precipitation, along with the physicochemical properties of water, which can support bacterial survival. Human activities, particularly agricultural and urban practices, contribute contaminants through runoff, fecal waste, and untreated wastewater. Understanding how these factors influence the persistence and spread of *Salmonella* in aquatic environments is essential for developing effective monitoring and mitigation strategies to reduce public health risks.

#### 3.3.1. Influence of Temperature on *Salmonella* Survival and Growth

Temperature is a critical factor for the proliferation of *Salmonella* in water bodies. Studies show a marked increase in *Salmonella* during warmer seasons, such as summer, when higher temperatures favor the survival and multiplication of pathogenic bacteria in surface waters [81,83]. Most *Salmonella* serotypes can grow within a temperature range of 5 °C to 47 °C, with an optimal range between 35 °C and 37 °C. However, growth is significantly reduced below 15 °C and is completely inhibited at temperatures below 7 °C [96]. Some strains can survive extreme temperatures, growing at as low as 2 °C and up to 54 °C [97,98,99].

Climate change is an additional factor contributing to the emergence of infectious diseases, including salmonellosis. Rising ambient temperatures correlate positively with the rise in gastrointestinal infections caused by *Salmonella* [100], as higher temperatures accelerate its reproduction and dissemination [12]. A linear relationship between salmonellosis rates and average temperatures over the previous week or month, with peaks occurring in summer [22]. An example of *Salmonella* growth capacity is observed in *S.* Typhimurium, whose generation time at 25 °C in enteral diets varies between 21 and 34.8 min, with a maximum growth rate (µmax) of 1.28 to 1.95 h^−1^ [101]. Under optimal conditions, the bacterial population can increase by 5 to 6 logarithmic cycles within 14 to 24 h [101].

#### 3.3.2. Precipitation and Its Impact on *Salmonella* Dispersal

Precipitation plays a crucial role in the contamination of water bodies with *Salmonella* [102]. Heavy rainfall generates runoff that transports fecal matter and contaminated sediments from agricultural and urban areas into rivers, lakes, and beaches, increasing microbial loads in the water [103]. Studies have found that the influence of extreme weather events on the incidence of salmonellosis is not immediate [22]; instead, there is typically a delay of 2 to 4 weeks between a high precipitation event and an increase in reported cases [22,104,105].

Drought conditions can also favor *Salmonella* persistence in water bodies. Reduced flow and evaporation concentrate contaminants in stagnant waters, increasing exposure risks [106]. In temperate regions, variations in water temperature influence the hydrodynamic distribution of microorganisms [107]. During summer, lakes generally stratify, with warmer water at the surface, but heavy rainfall events can disrupt this stratification (destratification) and redistribute pathogens in the water [108,109].

#### 3.3.3. Physicochemical Properties of Water and *Salmonella* Persistence

The physicochemical properties of water, such as pH, nutrient concentration, and dissolved oxygen levels, are key determinants in *Salmonella* survival [110,111,112]. This bacterium can grow within a pH range of 4 to 9, with an optimal pH between 6.5 and 7.5 [96]. Growth is favored in waters with moderate to high nutrient levels and warm temperatures [12]. Dissolved oxygen levels also influence *Salmonella* presence [110,111,112]. As a facultative anaerobe, *Salmonella* can survive in low-oxygen environments [113] and in waters with high organic matter content [114], where contaminated waste and sediments can act as reservoirs for the pathogen. In recreational water bodies such as beaches and lagoons, water quality can be compromised by factors like salinity and seasonal temperature variations [18].

#### 3.3.4. Human Activities and *Salmonella* Contamination in Water

Agricultural, urban, and livestock practices are key sources of *Salmonella* contamination in water bodies [115]. The use of manure as fertilizer in agricultural areas and runoff from contaminated fields introduce pathogens into surface water. Additionally, *Salmonella* in manure can survive for up to 231 days [116], posing a significant risk to agricultural production if carried by rainwater.

In urban areas, untreated or inadequately treated wastewater discharge is a major source of contamination [117]. The presence of *Salmonella* has been reported in treated effluents, with concentrations reaching up to 2.7 × 10^2^ CFU/100 mL [118,119]. Although modern wastewater treatment methods reduce bacterial loads, they do not completely eliminate *Salmonella*, posing a risk if treated water is reused for irrigation or discharged into surface waters [18].

Recreational activities also increase the risk of *Salmonella* transmission, as contact with contaminated water in beaches, lagoons, and rivers can be a source of infection [120]. Outbreaks of salmonellosis have been linked to the use of contaminated water in food production, such as papaya and cantaloupe [22], highlighting the importance of monitoring water quality in the food supply chain.

### 3.4. Isolation, Identification, and Serotyping of Salmonella

The isolation of *Salmonella* spp. from water samples was carried out using selective culture methodologies, enrichment, and biochemical and serological confirmation. In most of the studies analyzed, water samples were filtered using 0.45 µm membranes [23,66,67,68,69,87], although some studies employed alternative methods such as ultrafiltration or centrifugation at 4500 rpm [86], allowing for the concentration of the microorganism before the cultivation process.

For selective enrichment, specific broths such as Selenite-F [14,81], Rappaport-Vassiliadis (RV) [16,68,83], and Müller-Kauffmann Tetrathionate were used [16,66,83], incubated at temperatures between 35 and 42 °C [68,69,81,90] for 18–24 h [16,23,68,69,90]. Subsequently, the samples were plated on selective and differential media, with Xylose Lysine Deoxycholate (XLD) agar [17,85], Salmonella-Shigella (SS) [66,68,81,85,87], Brilliant Green [66,88,90,121], Bismuth Sulfite [67,88,90,121], and Hektoen Enteric [16,17,67,83] being the most used. *Salmonella* suspect colonies were characterized by black centers on differential media due to hydrogen sulfide (H₂S) production.

For the confirmation of isolates, standard biochemical tests were performed, including Triple Sugar Iron (TSI) [16,66,83,93] agar, Lysine Iron (LIA) [16,83,93], motility and indole test (SIM) [16,83], and the Indole, Methyl Red, Voges-Proskauer, and Citrate (IMViC) [67,93] series is commonly used for the identification of Enterobacteriaceae. Additionally, some studies implemented PCR for the *invA* gene as a complementary method for molecular identification [6,69,78,87].

Confirmed isolates were serotyped using the Kauffmann-White-Le Minor scheme [71,85] through agglutination tests with O and H antisera, enabling the identification of various serovars of public health importance, such as *S*. Typhimurium, *S*. Enteritidis, *S*. Panama, *S.* Anatum, and *S*. Newport. In some studies, serotyping was complemented with genotyping techniques such as Pulsed-Field Gel Electrophoresis (PEFG) or Whole Genome Sequencing (WGS) [78].

### 3.5. Antimicrobial Susceptibility of Salmonella

In addition to the identification of *Salmonella* serotype for understanding its epidemiological role, host specificity, and potential impact on public health, evaluating its antimicrobial susceptibility is critical, as water bodies can serve as reservoirs for antibiotic-resistant strains. The increasing prevalence of antimicrobial resistance among *Salmonella* isolates raises concerns about the role of contaminated water in the dissemination of resistant bacteria, which could compromise treatment options for both human and animal infections.

In the reviewed articles, antimicrobial resistance was primarily assessed using the disk diffusion method (Kirby-Bauer) [17,69], following the guidelines of the Clinical and Laboratory Standards Institute (CLSI) [6,10,16,68,69,71,85,92]. The tested antibiotics covered multiple classes, including beta-lactams (ampicillin, ceftriaxone, cefotaxime) [6,10,14,16,17,67,68,69,71,83,85,92], quinolones (ciprofloxacin, nalidixic acid) [6,10,14,16,17,68,69,71,83,85,92], sulfonamides (trimethoprim-sulfamethoxazole) [6,14,68,69,71,78,92], phenicols (chloramphenicol, florfenicol) [10,14,16,68,69,83], tetracyclines (tetracycline) [6,10,68,69,71,78,83,85], aminoglycosides (gentamicin) [14,16,83,92], nitrofuran derivatives (nitrofurantoin) [14,83], and polymyxins (colistin) [6,68,71]. Several of these antibiotics are used to treat both human and animal infections, such as chloramphenicol, florfenicol, gentamicin, ampicillin, and tetracycline [122,123], as well as colistin, an antibiotic of last resort for multi-resistant organisms (MDR) in humans [124], and florfenicol, which is used in aquaculture [125].

Results show that MDR isolates, defined as resistant to three or more antibiotic classes, are commonly found. Resistance to ampicillin, ceftriaxone, ciprofloxacin, chloramphenicol [6,10,14,16,17,67,68,69,71,83,85,92], and tetracycline [6,10,68,69,71,78,83,85] was the most frequently reported. However, resistance patterns varied across the reviewed studies, with some reporting higher resistance to beta-lactams and tetracyclines, while others described predominant resistance to quinolones and third-generation cephalosporins (Table S1).

## 4. Conclusions

The presence of *Salmonella* in water bodies across South America represents a significant challenge for both public health and the integrity of aquatic ecosystems. This pathogen is widely found in aquatic environments; its survival and proliferation are influenced by pollution, climate variability, and the physicochemical characteristics of water. Key contributing factors include the discharge of untreated wastewater, agricultural runoff, and elevated temperatures, which prolong *Salmonella* survival and facilitate its dissemination. Moreover, the rising incidence of multidrug-resistant strains underscores the urgency of addressing this issue from a comprehensive perspective.

The analysis of geographical distribution and predominant serovars in various countries highlights the diversity and complexity of contamination sources. Countries such as Argentina, Brazil, Chile, and Colombia exhibit specific patterns that reflect the interactions between anthropogenic activities, environmental conditions, and water management systems.

Among the most frequent serovars are *S.* Enteritidis and *S.* Typhimurium, which are widely associated with human and animal infections. In Argentina, *S.* Enteritidis predominates in irrigation systems and agricultural areas, while in Brazil, *S.* Typhimurium is prominent in aquaculture environments and wastewater. In Chile, *S.* Typhi and *S.* Panama have been identified in rivers used for irrigation and consumption, whereas in Colombia, contamination by *Salmonella* spp. in the Bogotá River and Atlantic beaches reflects the impact of untreated wastewater. Notably, the presence of less common serovars, such as Dublin, Paratyphi B, and those belonging to subspecies such as diarizonae and houtenae, demonstrates the influence of specific environmental and biological factors on the dynamics of this pathogen. This diversity of serovars, along with their adaptive capacity, emphasizes the need to implement more effective and continuous monitoring and control strategies.

The control of *Salmonella* in water bodies requires a comprehensive approach that combines prevention, monitoring, and mitigation strategies. At the preventive level, it is essential to improve wastewater treatment through advanced disinfection, regulate the use of antibiotics in livestock and aquaculture, and reduce agricultural runoff that can introduce resistant bacteria into water sources. The implementation of best practices in manure and fertilizer management, along with the protection of ecosystems such as wetlands and riparian forests, can also help limit the spread of *Salmonella* in the environment.

On the other hand, epidemiological surveillance and microbiological monitoring are key tools for early detection and rapid response to *Salmonella* outbreaks in water. The use of techniques such as real-time PCR and genomic sequencing allows for more precise identification of pathogenic strains and their antimicrobial resistance profiles. Additionally, public policies focused on water quality regulation, the application of the One Health approach, and improving access to sanitation in rural communities can significantly contribute to reducing the impact of *Salmonella* on public health and aquatic ecosystems.

Despite advances in understanding *Salmonella* contamination in South America, research and policy gaps remain. Long-term surveillance programs integrating climatic, hydrological, and microbiological data are needed to improve contamination predictions. At the policy level, stricter water quality regulations and enhanced transnational cooperation are essential for unified monitoring frameworks. The development of national groundwater monitoring networks in Argentina, Brazil, and Colombia provides a foundation for expanding standardized surveillance and contamination control. Strengthening policies on wastewater treatment and agricultural runoff management can further reduce *Salmonella* introduction into water systems.

Emerging technologies offer promising solutions for improving surveillance and outbreak detection. Early warning systems using remote sensing and machine learning could enhance real-time monitoring of water bodies, enabling faster responses. Additionally, whole genome sequencing (WGS) and metagenomics can provide valuable insights into *Salmonella’s* adaptability and resistance. Digital tools, such as web-based monitoring platforms and automated data processing systems, can improve data accessibility and analysis, ensuring a more proactive approach to contamination control.

Community-driven mitigation is also crucial. Public education on sanitation, food safety, and water conservation can help prevent infections, while citizen science programs involving local communities in water quality monitoring can enhance surveillance efforts. Strengthening clean water infrastructure, particularly in rural areas, is key to reducing waterborne disease risks.

Combining scientific research, policy development, and community action will be key to managing *Salmonella* contamination sustainably and protecting public health and aquatic ecosystems.

## Data Availability

No new data were created or analyzed in this study.

## Supplementary Materials

The following supporting information can be downloaded at: https://www.mdpi.com/article/10.3390/microorganisms13030489/s1, Table S1: Summary of *Salmonella* presence in South American water bodies. This table highlights the diversity of species and serovars, as well as their geographical distribution, including the number of confirmed *Salmonella* samples and the date of collection.

## Abbreviations

SC1	Search Component 1
SC2	Search Component 2
SC3	Search Component 3
RM	Metropolitan Region
MDR	multi-resistant organisms
